# Correction: Study on the inflammatory response of PMMA/polystyrene/silica nanocomposite membranes for drug delivery and dental applications

**DOI:** 10.1371/journal.pone.0215632

**Published:** 2019-04-15

**Authors:** S. Shanmuga Sundar, N. Kannan, E. Sundaravadivel, Sarang Zsolt, K. S. Mukunthan, J. Manokaran, J. Narendranath, V. P. Kamalakannan, N. P. Kavitha, N. V. Prabhu, N. Balasubramanian

The first, ninth, and tenth authors’ names are spelled incorrectly. The first author’s correct name is: S. Shanmuga Sundar. The ninth author’s correct name is: N. P. Kavitha. The tenth author’s correct name is: N. V. Prabhu. The correct citation is: Shanmuga Sundar S, Kannan N, Sundaravadivel E, Zsolt S, Mukunthan KS, Manokaran J, et al. (2019) Study on the inflammatory response of PMMA/polystyrene/silica nanocomposite membranes for drug delivery and dental applications. PLoS ONE 14(3): e0209948. https://doi.org/10.1371/journal.pone.0209948
